# Thymoquinone-PLGA-PVA Nanoparticles Ameliorate Bleomycin-Induced Pulmonary Fibrosis in Rats via Regulation of Inflammatory Cytokines and iNOS Signaling

**DOI:** 10.3390/ani9110951

**Published:** 2019-11-11

**Authors:** Sultan A. M. Saghir, Naif A. Al-Gabri, Asmaa F. Khafaga, Nahla H. El-shaer, Khaled A. Alhumaidh, Mohamed F. Elsadek, Badreldin M. Ahmed, Daniyah M. Alkhawtani, Mohamed E. Abd El-Hack

**Affiliations:** 1 Department of Medical Analysis, Princess Aisha Bint Al-Hussein College of Nursing and Medical Sciences, Al-Hussein Bin Talal University, Ma‘an 71111, Jordan; 2 Department of Pharmacology, School of Pharmaceutical Sciences, Universiti Sains Malaysia, Penang 11800, Malaysia; 3 Department of Pathology, Faculty of Veterinary Medicine, Thamar University, Dhamar 2153, Yemen; naifaljabry@yahoo.com; 4 Department of Pathology, Faculty of Veterinary Medicine, Alexandria University, Edfina 22758, Egypt; 5 Zoology Department, Faculty of Science, Zagazig University, Zagazig 44511, Egypt; nhelshaer@zu.edu.eg; 6 Department of Pharmacology and Toxicology, Faculty of Pharmacy, Kalamoon University, Damascus 222, Syria; alhmudh@yahoo.com; 7 Department of Community Health Sciences, College of Applied Medical Sciences, King Saud University, Riyadh 11362, Saudi Arabia; mfbadr@ksu.edu.sa (M.F.E.); Badreldin222@gmail.com (B.M.A.); dkhawtani@gmail.com (D.M.A.); 8 Department of Nutrition and Food Science, Helwan University, Helwan 11795, Egypt; 9 Poultry Department, Faculty of Agriculture, Zagazig University, Zagazig 44511, Egypt; dr.mohamed.e.abdalhaq@gmail.com

**Keywords:** pulmonary fibrosis, PLGA, thymoquinone, IL 10, TGF-β1, iNOS, ultrastructure

## Abstract

**Simple Summary:**

In this study, we evaluated the role of thymoquinone-PLGA-PVA nanoparticles (TQ-PLGA-PVA-NPs) in the amelioration of lung fibrosis induced experimentally in rats. Evaluation was performed via estimation of inflammatory cytokines in addition to the histopathological, immunohistochemical, and ultrastructural examination of lung tissues. Results illustrated the promising ameliorative role of TQ-PLGA-PVA-NPs.

**Abstract:**

Pulmonary fibrosis is considered one of the most chronic interstitial illnesses which are not easily treated. thymoquinone’s (TQ) benefits are still partly problematic due to poor water solubility; therefore, it was loaded onto PLGA-PVA carriers. This study aimed to evaluate the potential effect of TQ-PLGA-PVA nanoparticles (TQ-PLGA-PVA-NPs) on pulmonary fibrosis induced by bleomycin in albino rats. Forty male rats were randomized into four groups. The first group served as the control group; the second and the third groups received bleomycin intratracheally, whereas the third group received TQ-PLGA-PVA-NPs after 4 weeks from bleomycin administration. The fourth group was administrated TQ-PLGA-PVA-NPs alone. The designed nanoparticles appeared around 20 nm size (10–30 nm), had a spherical shape, and had 80% encapsulation efficiency. The histological examination of rats simultaneously treated with TQ-PLGA-PVA-NPs and bleomycin revealed reduction in the thickness of the alveolar septa and improvement of the other lung structures, with the presence of lymphocytes admixed with exfoliated epithelium in a few lumina remaining. Ultrastructural findings revealed marked collagenolysis and the release of nanoparticles from ruptured pneumocytes within the alveolar septa after 14 days from TQ-PLGA-PVA-NPs administration. Very active pneumocyte types II were seen in the TQ-PLGA-PVANP group. Additionally, immunohistochemical expression of inducible nitric oxide (iNOS) and estimation of inflammatory cytokines in lung tissues including interleukin 10 (IL 10) and transforming growth factor-beta (TGF-β1) confirmed the antioxidant and anti-inflammatory effects of TQ-PLGA-PVANPs. The study concluded that TQ-PLGA-PVA-NPs could attenuate the bleomycin-induced pulmonary fibrosis, through the inhibition of lung inflammation and the suppression of bleomycin- induced oxidative stress.

## 1. Introduction

Bleomycin, an antibiotic drug belong to the glycopeptides group, is one of the cancer chemotherapeutic agents commonly used in the treatment of several types of malignancy, including lymphoma (Hodgkin’s and non-Hodgkin’s) and solid tumors, such as testicular cancer, ovarian cancer, and cervical cancer [1,2]. Despite its effectiveness in treating cancers, many undesirable and serious side effects present in about 10% of patients due to its prolonged usage [3,4]. Even in low concentrations, bleomycin has the capacity to cause pulmonary inflammation via the production of reactive oxygen species (ROS), which may sporadically cause the development of pulmonary fibrosis (PF) in young and old patients, but its occurrence is higher among the elderly [5]. PF is a common, chronic pathological injury of the lung interstitium characterized by exaggerated production and deposition of the extracellular matrix, including fibroblasts and myofibroblasts [6,7]. Reactive nitrogen species (RNS) work together with ROS to damage cells through causing injury to DNA, lipid peroxidation, modification of lung prostaglandin, and collagen production. Subsequently, inflammation and interruption of cytokines, activation of fibroblasts, and stimulation of collagen production develop, whereas degradation of collagen is disturbed [2]. Together, these factors can lead to the development of extracellular matrix proteins that deform and destroy the normal structure of the alveoli and lead to impairment of pulmonary function [5].

RNS, comprised of nitric oxide (NO), peroxynitrite (ONOO-), and nitrogen dioxide (NO_2_), highly contribute to respiratory tract pathogenesis [8,9,10]. NO, an endogenous, short-lived, potent oxidant that spontaneously diffuses within the cells, was found to be responsible about the cytotoxic pathogenesis of lung tissues [11,12]. Furthermore, fibrosing alveolitis [13], asthma [14], and bronchiectasis [15] are highly associated with increased levels of exhaled NO. As a result of oxidative stress, cytokines and inflammatory factors, including interleukin 10 (IL 10) and transforming growth factor-beta (TGF-β1) are highly released from damaged lung tissues and upregulated with resultant promotion of lung fibrosis [16,17]. TGF-β1 is an inflammatory mediator which is considered a hallmark in pulmonary fibrosis [18]. It is the main inducer for epithelial–mesenchymal transition in alveolar epithelial cells, in addition to its role in the trans-differentiation of quiescent fibroblasts into myofibroblasts, which contributes to the deposition of extra cellular matrix [7]. Interestingly, the production and activation of TGF-β1 is controlled by IL 10, which can, in turn, play a role in fibrosis’s attenuation [19].

Usually, PF shows less survival advantages and a limited treatable rate after diagnosis. Induction of lung fibrosis in animal models using bleomycine is one of the widely used models because of its high simillarity to human PF [20]. Currently, limited trials for treatment of pulmonary fibrosis are available [21,22,23], but the development of a successful antifibrotic drug is still one of the everyday challenges. Previous findings connected excess NO production with PF and guided us to assume that any strategy which minimizes NO production would have beneficial effects on PF induced by Bleomycin [24].

For thousands of years, many natural agents have been used successfully in the treatment of various medical problems because of their safety and strong potential as antioxidant and anti-inflammatory agents. Among such agents, thymoquinone (TQ) is always included. TQ is the bioactive constituent of *Nigella sativa* seeds. The anti-fibrotic properties of the phytochemical TQ, have been described previously in several investigations in the liver [25,26]. Recently, TQ was used experimentally to alleviate the PF via inhibition of oxidative stress, down-regulation of pro-fibrotic genes, and blocking of the nuclear factor (NF-κB) [27]. However, the effectiveness of TQ is usually limited by its poor solubility and bioavailability. Currently, the improvement of bioavailable TQ is focused on in many studies through the nanoencapsulation technique. In recent studies, poly(lactic-co-glycolic) acid (PLGA) nanoparticles were loaded with tobramycin for the treatment of *P. aeruginosa*-induced cystic fibrosis in a patient’s lung, which led to sustained release of tobramycin over two days [28,29]. Moreover, PLGA encapsulated with TQ nanoparticle showed good, sustained release characteristics, antioxidant potential, and anti-microbial activity [30]. Additionally, TQ-PLGA was investigated against MDA-MB-231 cancer cell growths; the authors concluded that TQ-PLGA is more powerful antioxidant than free TQ alone [31]. Hence, this study was designed to evaluate, for the first time, the potential anti-inflammatory and anti-fibrotic effect of TQ-PLGA/PVA nanoparticles against bleomycin-induced PF and their possible underlying mechanism/s in rats via evaluation of the histopathological, immunohistochemical, and ultrastructural lung pictures, and via assessment of some inflammatory cytokines’ profiles.

## 2. Materials and Methods

### 2.1. Drugs and Chemicals

Thymoquinone (2-isopropyl-5-methyl-1, 4-benzoquinone), as a yellow crystalline form, was purchased from Sigma Aldrich (St. Louis, MO, USA) (product number 274666 1 GM). PLGA copolymers and polyvinylalcohol (PVA) were purchased from Sigma (St. Louis, MO, USA; product number P2191). Bleomycin hydrochloride was purchased from a pharmacy (Pubchem^®^). IL 10 (catalog number: 4157) and TGF-β1 (Catalog number: K4343) ELISA kits were purchased from bio vision (BioVision^®^, 155 S Milpitas Blvd. Milpitas, CA 95035, USA).

### 2.2. TQ-PLGA-PVA Nano-Emulsion Designing

**Figure 1:**
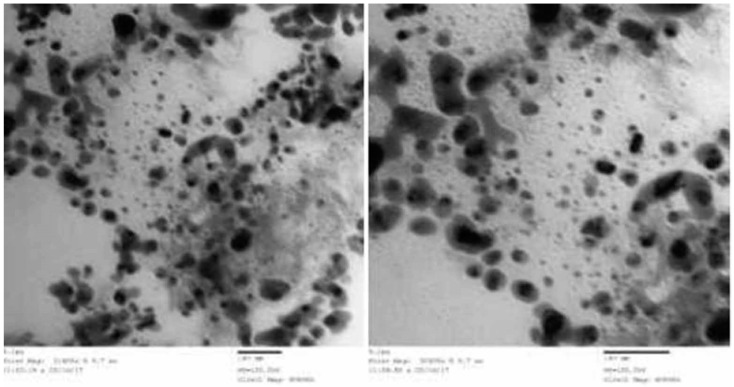
Electron micrograph of thymoquinone-PLGA nanoparticles suspension showing confirmation of size and shape. The designed nanoparticles appeared around 20 nm size and spherical shape. Scale bar = 50 nm (HV = 120.0 KV, Direct Mag: 40,000 X).

TQ was nano-formulated in PLGA nanoparticles via an emulsion technique using solid/oil/water methods with slight modifications [30,32]. Briefly, 80 mg of the PLGA powder) was dissolved in 2 mL of dicholoromethane (HPLC-grade) for 12 h as an oil phase in a room temperature to get a uniform solution. That was followed by the addition of 50 mg of TQ to the solution. The suspension was sonicated by sonication machine for 2 min to generate the solid/oil primary emulsion. Then the mixture (TQ/PLGA) was emulsified with an aqueous phase of 20 mL of saline with PVA 1% w/v to form a solid/oil/water emulsion by rotation in a magnetic stirrer at 400 rpm. The resulting suspension was ultra-sonicated (20 KH_2_) for 3 min to generate the final emulsion. Then, the organic solvent, which appeared in the above suspension, was evaporated by rotary vacuum evaporation at 50 °C. following that, the nanoparticles were collected by centrifugation at 10,000 r for 20 min at 4 °C. Finally, they were re-suspended in 2 mL of cryoprotectent solution (2% sucrose) and used for the experimental rats (Figure 1). After preparing the nanoparticles, the delivery was confirmed (size and shape of the particles), by using a transmission electron microscope at an accelerating voltage of 200 kV, in accordance with Vij et al. [33].

### 2.3. Animals

All precautions were taken to minimize number and suffering of animals. The minimum number of rats required to produce reliable scientific data was used. Forty male Wister albino rats, 8–10 weeks old, at 200 ± 25 g body weight, were obtained from the animal house of the Faculty of Veterinary Medicine, Zagazig University, Egypt. Rats were kept in metal cages under controlled environmental conditions (24 ± 3 °C temperature, 50–55% RH, and 12 h light/12 h dark cycle) for a one week acclimatization. Rats were fed on a standard pellet ration (El-Nasr Chemical Company, Cairo, Egypt), and they were allowed accesses to water ad libitum. All animals were managed according to Animal Ethical Committee of Faculty of Veterinary Medicine, Zagazig University, Egypt.

### 2.4. Experimental Design

The experimental rats were allocated into four equal groups (ten rats each). Rats in Group 1 were intratracheally (i.t.) instilled with single dose of saline 200 µL/rat and served as the control group. Rats in Groups 2 and 3 (bleomycin and bleomycin + TQ-PLGA-PVA-NPs, respectively) were i.t. instilled with single dose of bleomycin equal to 200 µL/rat (200 µL normal saline containing bleomycin at dose 5 mg/kg) to induce pulmonary fibrosis, as previously discussed by Harrison and Lazo [34]. After 4 weeks of bleomycin i.t. instillation, rats in Group 3 (bleomycin+ TQ-PLGA-PVA-NPs) were instilled with i.t. by 200 µL/rat of TQ-PLGA-PVA-NPs; and finally rats in Group 4 (TQ-PLGA-PVA-NPs) were instilled i.t. with 200 µL/rat of TQ-PLGA-PVA-NPs alone. Rats from all groups were anesthetized and euthanized 14 days post nano-installation. After euthanasia, rats were necropsied and grossly examined. Small specimens were collected from lungs, washed in PBS, and fixed in 10% neutral buffered formalin solution for further histopathologic and immunohistochemical examination. Other small specimens were rapidly kept in 2.5% glutaraldehyde for ultrastructure examination under transmission electron microscope, and the third parts of specimens were kept frozen for further estimation of tissue cytokines.

### 2.5. Induction of Lung Fibrosis

**Figure 2:**
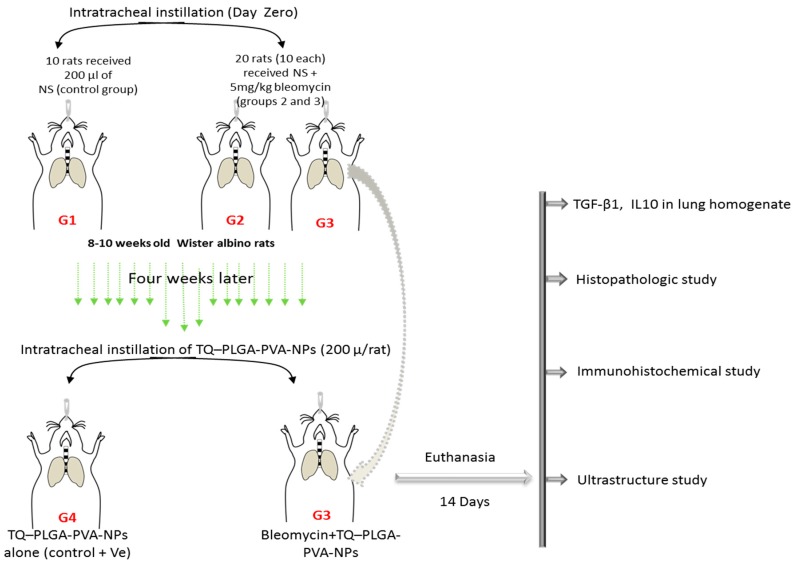
Schematic protocol for experimental procedures. Rats received intratracheal instillation with 200 µL of (Group 1) normal saline (NS); (Group 2) NS + bleomycin (5 mg/kg); (Group 3) NS + bleomycin (5 mg/kg) + TQ-PLGA-PVA-NPs (NPs—nanoparticles); (Group 4) NS+TQ-PLGA-PVA-NPs. Rats were euthanized 14 days post intratracheal instillation; lung specimens were collected for further evaluation of histopathological, immunohistochemical, and ultrastructural pictures, and for cytokines profiles. (Group 4) NS+TQ-PLGA-PVA-NPs;: thymoquinone poly lactic-co-glycolic acid (PLGA) nanoparticles.

Bleomycin-induced lung fibrosis was performed through a modified manner from the previous reports of Harrison and Lazo [34] and Al-Gabri et al., [35]. Briefly, thirty rats were anesthetized by intraperitoneal (i.p.) injection of xylazine and ketamine at a dose of 80 mg/kg and 12 mg/kg respectively, by using an insulin syringe [36]. The rat was fixed on their back, over a glass board with an angle of 70°. Then, 200 µL of normal saline alone (adminstrated to ten rats—served as control) or containing 5 mg/kg bleomycin (adminstrated for twenty rats—G2 and G3) were instilled into the rats’ tracheas by the using 3-gauge intravenous plastic needles fixed on insulin syringes, followed by 0.3 mL air boluses to allow the doses to reach and distribute to the distal portions of the airways and lungs. After intratracheal instillation, the rats were placed in a posterior vertical position and rotated for 1 min to distribute the instillation equally within the lungs. The schematic protocol is summarized in Figure 2.

### 2.6. Assessment of Levels of TGF-β1 and IL 10

Levels of interleukin 10 (IL 10) and transforming growth factor-β1 (TGF-β1) were measured in lung homogenate using a commercially available ELISA kit (BioVision^®^ rats ELISA kit; catelogue numbers: 4157 and K4343 respectively) (155 S Milpitas Blvd. Milpitas, CA 95035, USA) according to the manufacturers’ instructions. The sensitivity of the assay was 5.1 pg/mL. The samples were examined in duplicate, and the experiment was repeated at least three times.

### 2.7. Histopathologic Assessment

After euthanasia, rats’ lungs were surgically removed and flushed with phosphate buffer saline (PBS, pH 7.4). Small lung specimens were fixed in a 10%, neutrally-buffered formalin solution for at least 48 h. The fixed specimens were processed through the conventional paraffin embedding technique via dehydration in ascending grades of ethyl alcohol, clearing in three changes of xylene, and embedding in paraffin blocks at 65 °C. Four µm thick sections were stained routinely by Hematoxylin and Eosin (H&E) according to the method described by Bancroft and Gamble [37]. Additional sections were stained with Masson’s trichrome according to the method described by Crookham and Dapson [38] for clear demonstration of fibrous tissues. Representative micrographs of the sections were obtained with a digital camera (Leica EC3, Leica, Germany) connected to a microscope (Leica DM500, Leica Microsystems GmbH, Ernst-Leitz-Strasse 17-37, 35578 Wetzlar Germany). The severity of histopathological lesions was evaluated through semiquantitative scoring for five randomly selected fields (×100) from each lung sample, through assessment of the area percentage affected in the entire section as follows: 0 = absence of lesion, 1 = 5–25%, 2 = 26–50%, and 3 = ≥50%, as in Khafaga [39] and Al-Gabri [40]. In addition, the quantitative histomorphometric analysis of the area percentage of Masson’s trichrom, positive-stained fibros tissues was performed for lung tissues (5 random fields from each section) of the control and treated rats using Image (NIH, Bethesda, MD, USA).

### 2.8. Immunohistochemical and Histo-Morphometric Assessment

From each paraffin block, 4 μm thick sections were obtained, deparaffinized, and rehydrated in descending grades of ethanol. Antigen was retrieved using 0.01 mol/L citrate–buffered saline (pH 6.0) and quenched for endogenous peroxidase activity using 0.3% (v/v) H_2_O_2_ in phosphate–buffered saline. Following that, blocking against non-specific binding of the immunological reagents was performed via incubation with normal goat serum 10% (v/v) for 1 h. Lung sections were incubated overnight at 4 °C with iNOS rabbit polyclonal antibody (Bioss Inc, Cat.: bs-2072R, Boston, MA, USA) with dilution 1:300. After washing with PBS, the sections were incubated with biotin-conjugated goat anti-mouse IgG antiserum (Histofine kit, Nichirei Corporation, Tokyo, Japan) for 60 min, then washed in PBS, and incubated with streptavidin-peroxidase conjugate (Histofine kit, Nichirei Corporation, Tokyo, Japan) for 30 min. The streptavidin-biotin complex was visualized with 3,3′-diaminobenzidine tetrahydrochloride (DAB)-H_2_O_2_ solution, pH 7.0, for 3 min. Then, sections were counterstained using Mayer’s hematoxylin solution [41]. For the quantitative histo-morphometric analysis, original micrographs were obtained from immunostained slides (5 random fields from each section; ×100) using a digital camera (Leica EC3, Leica, Germany) connected to a microscope (Leica DM500, Leica Microsystems GmbH, Ernst-Leitz-Strasse 17-37, 35578 Wetzlar Germany). The area percentage of iNOS immune expression was measured in lung tissues of control and treated rats using Image (NIH, Bethesda, MD, USA) [42].

### 2.9. Ultrastructure Assessment

For ultrastructure examination, lung specimens were trimmed into fine sections by a sharp blade, and immediately fixed in 2.5% glutaraldehyde at pH 7.2 for 4 h; then, transferred to 1.33% osmium tetroxide overnight at 4 degrees. Following that, specimens were prepared as previously described by Hayat [43]. Briefly, tissues were dehydrated, cleared, and embedded in epoxy resin. Semi-thin sections were obtained by ultra-microtome, stained with toluidine blue, and evaluated by light microscope to detect lesions. Ultra-thin sections were obtained by ultra-microtome, uploaded on the grid, stained with lead citrate and uranium, and finally, evaluated by transmission electron microscopy (JEOL JEM-1230).

### 2.10. Statistical Analysis

The laboratory data concerning levels of IL 10 and TGF-β1were statistically analyzed using one-way analysis of variance (ANOVA) on the Statistical Analysis System software (SAS Institute Inc Cary: 100 SAS Campus Dr, Cary, NC, USA) [44] followed by Duncan’s multiple range test. Data are presented as means ± standard errors. *p* < 0.05 was considered statistically different. The semi-quantitative scoring of lung injury parameters and the quantitative analyses of the percentage areas of iNOS immune expression and Masson’s trichrom-stained fibrous tissues were subjected to nonparametric analysis using Kruskal–Wallis tests to assess the significance between mean scores obtained from Wilcoxon rank sum tests.

## 3. Results

### 3.1. Characterization of Designed Nanoparticle

The morphology of TQ-PLGA-PVA-NPs was evaluated using high resolution transmission electron microscope (JEOL JEM-2100, Tokyo, Japan). Nanoparticles appear in a suspension form, with a spherical shape, are transparent to turbid white color, and have a particle size around 20 nm (10–30 nm) (Figure 1). In addition, the encapsulation efficiency of the prepared particles was calculated to reach 80% efficiency.

### 3.2. Gross Examination of Lung Tissues

**Figure 3:**
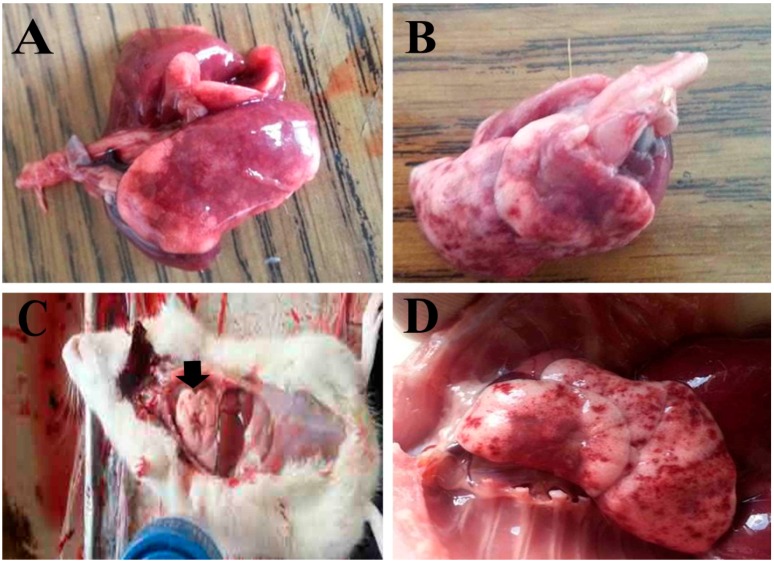
The effects of intratracheal administrations of bleomycin (**A**,**B**) or bleomycin+TQ-PLGA-PVA-NPs (**C**,**D**) on lung tissues of treated rats; (**A**,**B**) gross pneumonia (cornification and consolidation stage), depressed area, and compensatory emphysema in the periphery. (**C**,**D**) Similar gross lesions with less severity and incidence.

Lung tissues from bleomycin-treated rats showed gross pneumonia (cornification and consolidation stage), depressed area, and compensatory emphysema in the periphery. However, lung tissues from bleomycin + TQ-PLGA-PVA-NPs treated rats showed almost similar gross signes with less severity and incidence (Figure 3).

### 3.3. Levels of the Pulmonary TGF-β1 and IL 10

**Figure 4:**
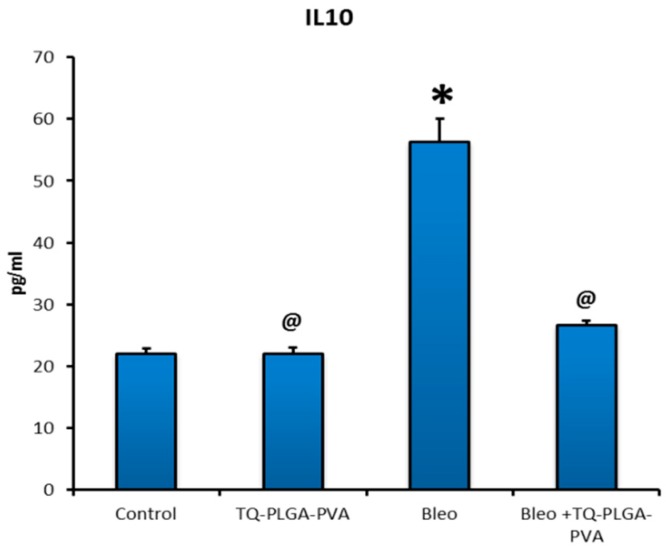
The effect of TQ-PLGA-PVA-NPs on levels of interleukin10 (IL 10) in bleomycin-induced pulmonary fibrosis in rats’ lungs. Results are presented as means ± SEs. Statistical analyses were achieved using one-way ANOVA followed by Duncan’s multiple range test; * *p* < 0.05 compared with the normal control. @ *p* < 0.05 compared with the bleomycin-treated rats.

**Figure 5:**
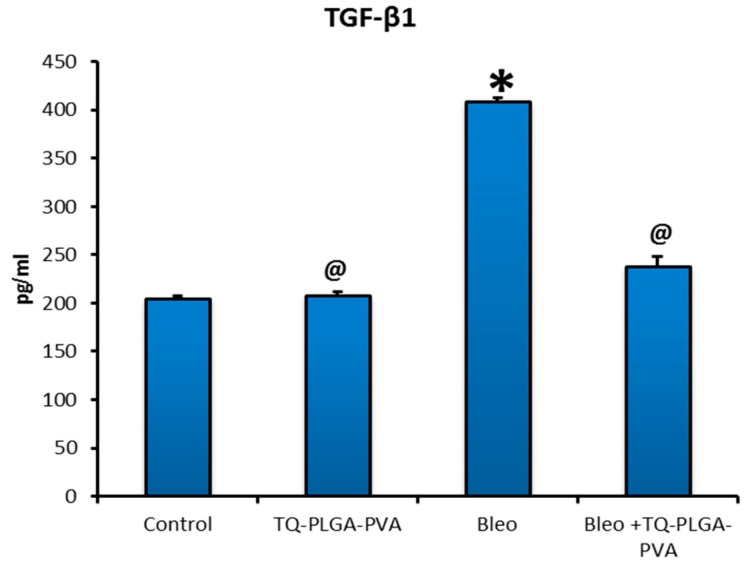
The effect of TQ-PLGA-PVA-NPs on levels of transforming growth factor beta 1 (TGF-β1) in bleomycin-induced pulmonary fibrosis in rats’ lungs. Results are presented as means ± SEs. Statistical analyses were achieved using one-way ANOVA, followed by Duncan’s multiple range test. * *p* < 0.05 compared with the normal control. @ *p* < 0.05 compared with the bleomycin-treated rats.

As shown in Figure 4, analysis of pulmonary levels of IL 10 in lung tissues homogenate of different groups revealed significant upregulation in bleomycin-treated rats (56.38 ± 3.67 pg/mL) compared to control rats (22.17 ± 0.68 pg/mL), while this level was reduced significantly in rats treated with bleomycin + TQ-PLGA-PVA-NPs (26.77 ± 0.53 pg/mL). On the other hand, the pulmonary level of IL 10 in TQ-PLGA-PVA-NPs treated rats did not show any significant difference (22.19 ± 0.80 pg/mL) compared to control rats. Regarding to TGF-β1, as illustrated in Figure 5, lung levels were increased significantly in bleomycin-treated rats (408.9 ± 30.21 pg/mL) compared with their levels in control and bleomycin + TQ-PLGA-PVA-NPs treated rats (208.2 ± 2.92 pg/mL and 238.1 ± 9.74 pg/mL, respectively). However, TQ-PLGA-PVA-NPs treated rats did not reveal significant changes (204.5 ± 2.54 pg/mL) in TGF-β1 levels compared to control rats.

### 3.4. Histopathological Findings

**Figure 6:**
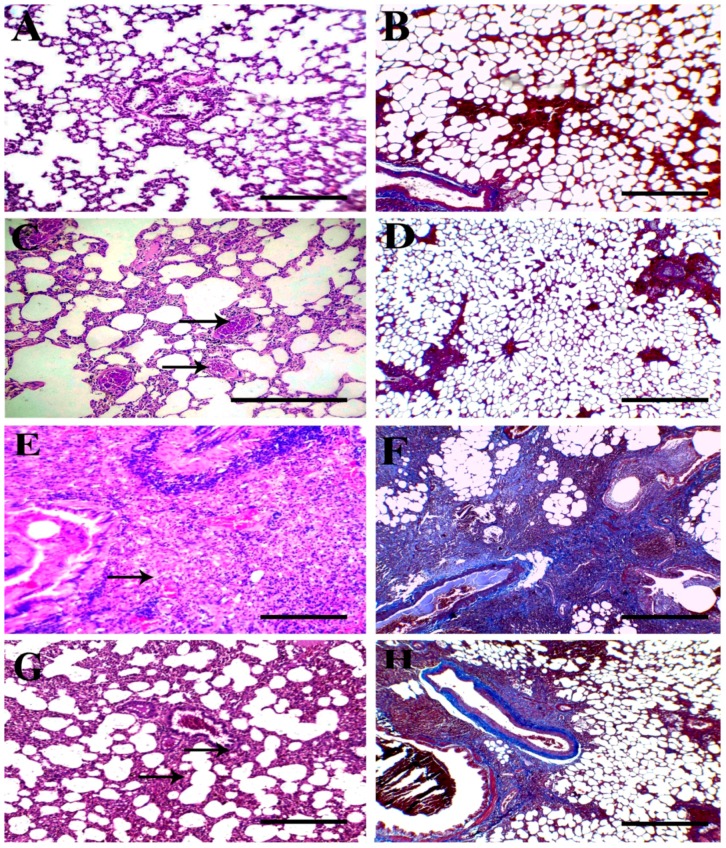
Representative photomicrograph of H&E and Masson’s trichrome (MT) stained sections from Wister rats’ lungs treated (via intratracheal administration) with bleomycin, TQ-PLGA-PVA-NPs, or their combination. (**A,B**) Lung sections from control rats showing normal histological architecture and normal limits of peribronchial collagen deposition. (**C,D**) Lung sections from TQ-PLGA-PVA-NP-treated rats showing slight thickening of interalveolar septa and congestion of interstitial blood capillaries (arrows). MT stained sections appeared without over collagen deposits around airways. (**E,F**) Lung sections from bleomycin-treated rats showing complete obliteration of pulmonary alveoli with proliferated spindle cells and variable amounts collagen fibers (arrows). The MT stained section revealed intense stainable blue collagen deposits. (**G,H**) Lung sections from bleomycin + TQ-PLGA-PVANP-treated rats showing partial improvement of lung tissues, with moderate infiltration of chronic mononuclear cells, predominantly lymphocytes, admixed with exfoliated epithelium (arrows). MT stained sections show moderate peribronchial and perivascular collagen depositions. Bar = 200 µm.

Examination of lung tissues from control rats revealed normal histological structures for alveoli, bronchi, and bronchioles, as well as the interalveolar septa and vasculature (Figure 6A). In addition, mild thickening of alveolar septa was reported in TQ-PLGA-PVA-NPs treated rats with mild congestion of interalveolar blood capillaries (Figure 6C). Examination of lung tissues from bleomycin-treated rats showed complete obliteration of the majority of lung alveoli due to severe infiltration of chronic inflammatory cells, predominantly fibroblasts and alveolar macrophages, with deposition of variable amount of collagen fibers in alveolar septa (Figure 6E). In addition, marked compensatory emphysema was described in neighboring alveoli to compensate the lost function of obliterated alveoli. Rats treated with bleomycin and TQ-PLGA-PVA-NPs showed moderate improvement for the alveolar septa structure with a moderate presence of chronic inflammatory cells, mainly lymphocytes admixed with exfoliated epithelium (Figure 6G). The adjacent alveoli showed lesser degrees of emphysema. The Masson’s trichrome stained sections revealed intense blue staining for collagen deposits in bleomycin-exposed rats (Figure 6F). However, moderate collagen depositions, particularly around the blood vessels and airways, were detected in the lungs of rats treated with bleomycin and TQ-PLGA-PVA-NPs (Figure 6H). Lung tissues from control and TQ-PLGA-PVA-NPs appeared without collagen deposits around airways (Figure 6B,D). Results obtained from the quantitative evaluation of Masson’s trichrome stained fibrous tissues (expressed as area percentages) are presented in Figure 8A; lung tissues from control and TQ-PLGA-PVA-NPs treated rats showed negative to mild depossition of fibrous tissue in the field evaluated. However, rats intoxicated with bleomycin expressed a marked increase in their area percentages of stained fibrous tissues. Cotreatment with TQ-PLGA-PVA-NPs induced significant reduction in collagen deposits. The histopathologic lesion score of different experimental groups was summarized in Table 1.

### 3.5. Immunohistochemical Findings

**Figure 7:**
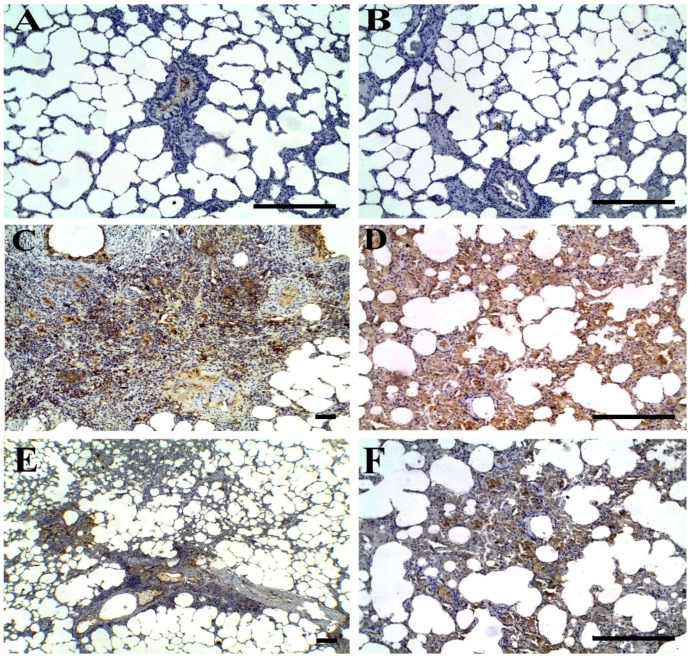
Representative photomicrographs of iNOS immunoreactivity in the lung tissue of Wister rats treated (via intratracheal instillation) with bleomycin, TQ-PLGA-PVA-NPs, or their combination; scale bar = 100 μm. Lung tissues from control (**A**) and TQ-PLGA nanoparticles treated rats (**B**) showed negative iNOS immune expression. Bleomycin-intoxicated rats (**C,D**) exhibited strong iNOS immunoreactivity in peribronchial (**C**) and interalveolar (**D**) (higher magnification) tissues. Lung tissues from rats co-treated with TQ-PLGA-PVA-NPs (**E,F**) show mild to moderate iNOS immunoreactivity in peribronchial (**E**) and interalveolar (**F**) (higher magnification) tissues.

**Figure 8:**
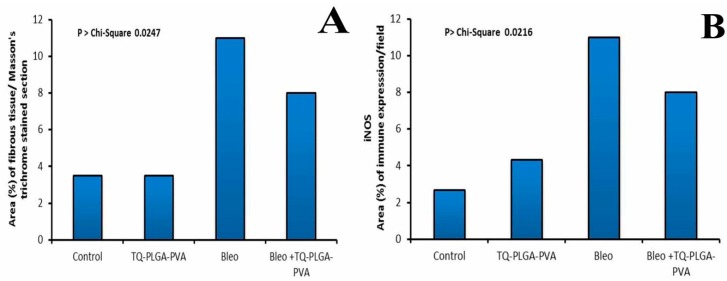
Area percentage of (**A**) Masson’s trichrom stained fibrous tissues, and (**B**) iNOS immune expression in lung tissues from control and treated groups; all scores were subjected to nonparametric analysis using the Kruskal–Wallis test to assess the significance between mean scores obtained from the Wilcoxon rank sum test (*p* > Chi-square < 0.0247 for (**A**); *p* > Chi-square < 0.0216 for (**B**)).

As shown in Figure 7, lung tissues from control (Figure 7A) and TQ-PLGA-PVA-NP-treated rats (Figure 7B) showed nearly negative immune expression of iNOS. However, rats intoxicated with bleomycin expressed a marked increase in iNOS immunoreactivity within the peribronchial (Figure 7C) and interalveolar regions (Figure 7D). Cotreatment with TQ-PLGA-PVA-NPs induced significant reduction in iNOS immunoexpression within the thickned peribronchial (Figure 7E) and interalveolar regions (Figure 7F). Non-parametric analysis of area percentage of iNOS immune expression in lung tissues from control and treated groups is illustrated in Figure 8B.

### 3.6. Ultrastructure Findings

**Figure 9:**
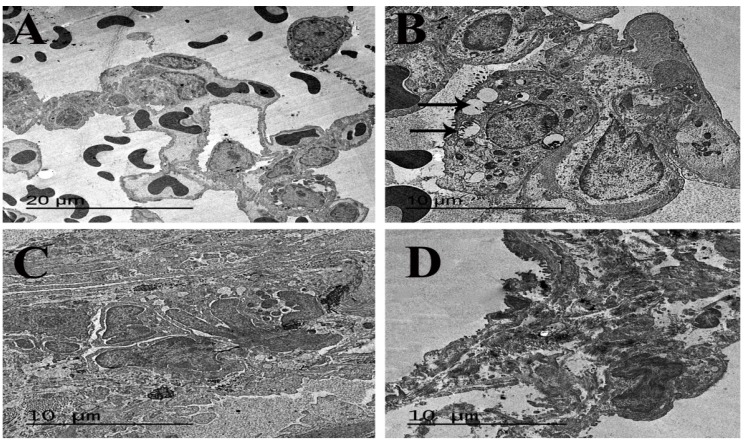
Representative electron micrograph for ultra-thin lung sections from Wister rats treated (via intratracheal instillation) with bleomycin, TQ-PLGA-PVA-NPs, or their combination. (**A**) Lung sections from control rats showing apparently normal pneumocytes and blood air barriers. (**B**) Lung sections from TQ-PLGA-PVA-NPs- treated rats showing active pneumocyte type II with prominent lamellar bodies with or without surfactant lamella (arrows). (**C**) Lung sections from bleomycin- treated rats showing the presence of an excessive amount of collagen fibers intermingled interstitially between proliferated cells. (**D**) Lung sections from bleomycin+TQ-PLGA-PVA-NPs treated rats showing collagenolysis and release of nanoparticles from ruptures pneumocytes within the alveolar septa (bar = 20 µm for (**A**); bar = 10 µm for (**B**–**D**)).

Examination of the lung tissues from the control rat revealed normal structure of pneumocytes and blood air barriers (Figure 9A). Moreover, very active pneumocyte types II with prominent lamellar bodies with or without surfactant lamella were seen in TQ-PLGA-PVA-NPs treated rat lungs (Figure 9B). Bleomycin-treated rats revealed the presence of intense types of collagen fibers, which were intermingled between interstitially proliferated cells (Figure 9C). Meanwhile, ultrastructural examination of lung tissues from rats that received bleomycin and TQ-PLGA-PVA-NPs showed marked collagenolysis and release of nanoparticles from ruptures pneumocytes within the alveolar septa (Figure 9D).

## 4. Discussion

Thymoquinone (TQ), the major active component of *Nigella sativa*, has been traditionally used for several medicinal purposes, including antioxidant [45], anti-inflammatory [46], hypocholestromic [47], anticancerous [48], hepato-protective [49], antmicrobial [50], and immunomodulatory [51]. However, the effectiveness of TQ is limited by its poor solubility and bioavailability. Recently, the improvement of bioavailablity was focused on by many researchers through the nanoencapsulation technique. In the present study, TQ-loaded PLGA nanoparticle were prepared by using of PVA as a stabilizing agent. The aim of the present study was to point out the therapeutic role of TQ-PLGA-PVA-NPs against bleomycin-induced pulmonary fibrosis. Histopathologic findings confirmed that pulmonary tissues from bleomycin-treated rats suffered complete obstruction of pulmonary alveoli with proliferated spindle cells and collagen fiber deposition. Intratracheal administration of TQ-PLGA-PVA-NPs concurrently with bleomycin led to partial resolution and remodeling of lung tissues and alveolar septa. These results are in agreement with those obtained by Abidi et al., [52]; they confirmed the effectiveness of bleomycin in the induction of pulmonary fibrosis and the initiation of prominent inflammatory events and TGF-β1 distribution in inflammatory infiltrates of experimental rats’ lungs. However, rats treated with *Nigella sativa* oils showed reduced inflammatory index and pulmonary fibrosis. Pulmonary fibrosis induced by bleomycin could be attributed to several mechanisms, such as oxidative damage, failure of the deactivating enzyme, and elaboration of inflammatory cytokines [2,53]. Hosseini et al., [54] concluded the role of the phytomedicinal plant extracts in amelioratation of bleomycin-induced lung fibrosis through several mechanisms, such as inhibition of inflammatory cytokines, oxidative stress, and fibrotic markers [54]. Specifically, many previous reports confirmed the characteristic effects of TQ against hepatic [25,26] and pulmonary fibrosis via down-regulation of pro-fibrotic genes, oxidative stress, and inhibition of NF-B [55].

TGF- β 1 is a multifunctional cytokine, which contributes to fibrosis mainly through the transformation of fibroblasts into myofibroblasts and promotion of collagen deposition [56,57]. It is well-established that the upregulation of TGF-β1 is associated with chronic fibrotic and inflammatory conditions [58]. Several recent works concluded that activation of TGF-β1 was caused by ROS [58], while the exposure of epithelial cells and fibroblasts to TGF-β1 was able to enhance the oxidative stress–mediated cytotoxicity and collagen synthesis and deposition. In our study, treatment with TQ-PLGA-PVA-NPs blocked TGF-β1-enhanced collagen deposition, where significant upregulation of lung levels of TGF-β1 was noticed in bleomycin-treated rats compared to bleomycin+TQ-PLGA-PVA-NPs treated rats. This result strongly suggests that TQ-PLGA-PVA-NPs have a role in suppression of bleomycin-induced early inflammation. These results were in line with the results obtained previously by Kurosaki et al. [55,59,60,61,62], where they found that TQ induced down-regulation for the inflammatory cytokines. Contritely, IL 10 is a key word in regulation of the inflammatory conditions, which might resuld in further tissue injury; wherefore, blocking of IL 10 signaling could result in marked pathologic lesions or even death [63]. However, a high level of IL 10 is usually related to persistent chronic disease, such as fibrosis, so its blocking enhances the pathogen elimination [64]. Consistent to that, lung levels of IL 10 increased in bleomycin-treated rats (408.9 pg/mL); however, it was blocked significantly in bleomycin + TQ-PLGA-PVA-NP-treated rats (238.1 pg/mL), which supports the amelioration theory of TQ-PLGA-PVA-NPs against bleomycin-induced lung fibrosis through regulation of TGF-β1 and IL 10. Similarly, Nakagome et al. [19] concluded that IL 10 was able to suppresses the production of TGF-β1 in the lung with subsequent suppression of fibrosis.

According to the growing body of evidence, oxidative stress plays a critical role in lung fibrosis [64,65]. In the lung tissues, various oxidants and pollutants favor the fibrotic interstitial lung reactions, where they are able to upregulate the oxidant production, and in turn, activate the inflammatory cells to generate free radicals [64]. Furthermore, these oxidants may prompt the production of the reactive oxygen species (ROS)—hydroxyl radicals, hydrogen peroxide, and the superoxide radical. Superoxide may react with nitric oxide (NO), whereas NO is principally produced by the inducible form of nitric oxide synthase (iNOS, NOS_2_) in the lung, particularly during inflammation [66]. In the current study, immunohistochemical expression of iNOS was extensive in bleomycin-intoxicated rats, suggesting a strong oxidative effect of bleomycin, and a lesser one of bleomycin+TQ-PLGA-PVA-NPs. This finding can provide an acceptable view about the antioxidant effect of TQ-PLGA-PVA-NPs.

Consistently, the ultrastructure study for lung tissues from bleomycin-treated rats confirmed the presence of excessive multiple bundles of collagen fibers intermingled between the interstitially proliferated cells, which is consistent with the histopathologic results. However, the electron micrograph obtained of the alveolar septa of bleomycin + TQ-PLGA-PVA-NPs treated rats revealed normal pulmonary structures with marked collagenolysis and the release of nanoparticles from ruptured pneumocytes within the alveolar septa. These findings suggested that the TQ-PLGA-PVA nano-formulation possesses ameliorating properties against the toxic and fibrotic effect of bleomycin. These findings are in complete agreement with the findings noted by Al-Gabri et al. [40] and Metwally and Al-Gabri [67] after treatment of lipopolysaccharide-induced lung injury with crude TQ or surfactant. The antifibrotic potential of TQ-PLGA-PVA-NPs may be attributed to the sustained release of nanoparticles within the lung tissues, which was noted, in our study, after 14 days of intratracheal instillation. The sustained release properties was previously discussed by Nallamuthu et al., [30] and Zolnik et al. [68], who showed that the sustained release of TQ reached 75% and 54% for intestinal and gastric conditions after 7 days.

## 5. Conclusions

According to the results, it could be concluded that TQ uploded to PLGA-PVA nanoparticles is a promising formula which could be used to ameliorate bleomycin-induced pulmonary fibrosis via regulation of TGF-β1 and IL 10 and via downregulation of iNOS in lung tissues. Further studies are needed to compare the antifibrotic effect of thymoquinone and its nano-formulation, and to compare different polymers which are used as viable materials for NP drug delivery systems, such as PLGA, PLG, PLA, and chitosan.

## Figures and Tables

**Table 1:** The severity of histopathological lesions recorded in the lung tissues of male Wister albino rats treated with bleomycin, TQ-PLGA nanoparticles, or their combination.

Scored Lesions	Experiment Groups			
	Bleomycin	Bleomycin + TQ-PLGA-PVA-NPs	TQ-PLGA-PVA-NPs	Control
Fibrosis	3	1	0	0
Focal pneumonic areas	3	1	0	0
Hyperplasia and hypertrophy of pneumocytes I & II	3	1	0	0
Thickening of alveoli septal	3	1	1	0
Atelectasis	2	0	1	0
Alveolitis	3	1	1	0
Compensatory emphysema	2	1	1	0

Number of examined rats = 10 rats/group. Number of examined fields (five fields/rat, ×100). The severity of a lesion was graded by estimating the percentage area affected in the entire section: 0 = absence of lesion, 1 = 5–25%, 2 = 26–50%, and 3 = ≥50%.
